# Assessing dengue control in Tokyo, 2014

**DOI:** 10.1371/journal.pntd.0007468

**Published:** 2019-06-21

**Authors:** Baoyin Yuan, Hyojung Lee, Hiroshi Nishiura

**Affiliations:** 1 Graduate School of Medicine, Hokkaido University, Sapporo-shi, Hokkaido, Japan; 2 CREST, Japan Science and Technology Agency, Kawaguchi, Saitama, Japan; University of Washington, UNITED STATES

## Abstract

**Background:**

In summer 2014, an autochthonous outbreak of dengue occurred in Tokyo, Japan, in which Yoyogi Park acted as the focal area of transmission. Recognizing the outbreak, concerted efforts were made to control viral spread, which included mosquito control, public announcement of the outbreak, and a total ban on entering the park. We sought to assess the effectiveness of these control measures.

**Methodology/Principal findings:**

We used a mathematical model to describe the transmission dynamics. Using dates of exposure and illness onset, we categorized cases into three groups according to the availability of these datasets. The infection process was parametrically modeled by generation, and convolution of the infection process and the incubation period was fitted to the data. By estimating the effective reproduction number, we determined that the effect of dengue risk communication together with mosquito control from 28 August 2014 was insufficiently large to lower the reproduction number to below 1. However, once Yoyogi Park was closed on 4 September, the value of the effective reproduction number began to fall below 1, and the associated relative reduction in the effective reproduction number was estimated to be 20%–60%. The mean incubation period was an estimated 5.8 days.

**Conclusions/Significance:**

Regardless of the assumed number of generations of cases, the combined effect of mosquito control, risk communication, and park closure appeared to be successful in interrupting the chain of dengue transmission in Tokyo.

## Author summary

Evaluating the interventions implemented during an outbreak of mosquito-borne disease is of utmost importance, offering lessons for future control strategies. By retrospectively analyzing data of the first autochthonous dengue epidemic of the 21st century in Tokyo, Japan, we assessed the effectiveness of the interventions. Once a dengue outbreak was confirmed in late August 2014, the government of Japan took drastic mosquito control measures, targeting both adults and larvae. News of the outbreak was also widely disseminated via mass media along with experts’ recommendations as to how people could avoid the risks of dengue infection. As the outbreak was not immediately controlled, the focal area of transmission, Yoyogi Park, was closed on 4 September. Using a mathematical model, we assessed how well dengue virus transmission was intervened in relation to the start times of interventions. As we incorporated precise timing into the model, we directly modeled the time of infection and accounted for the time delay from infection to illness onset. Thus, we revealed that mosquito control and risk communication measures alone could not interrupt the chain of transmission; however, adding park closure to these interventions was substantially effective in reducing the number of transmissions.

## Introduction

Dengue fever is a vector-borne viral disease, caused by dengue virus (DENV) and transmitted by *Aedes aegypti* and *Aedes albopictus* [1–4]. There are four antigenically related serotypes (DENV1 to 4), and first infection with one serotype is often self-limiting [5], eliciting specific acquired immunity. However, following primary infection with one serotype, infected and recovered individuals remain prone to secondary infection with other serotypes that could induce a clinically severe form of infection, including dengue hemorrhagic fever and dengue shock syndrome [6,7]. DENV infections are seen mostly in tropical and subtropical countries, but nonendemic areas in temperate regions are also at risk [5,8–11], owing partly to the changing ecological dynamics of vector abundance, perhaps induced by global warming [12] and an increased volume of international travel [13]. Although vaccination is partly underway in endemic countries, concrete plans for the prevention and specific treatment of dengue have yet to be fully established [7,14]. Moreover, the possible drawbacks of vaccination in low-endemicity settings remain a controversial issue [7].

Japan is in a temperate zone and dengue is not endemic in the country. However, Japan has experienced a steady increase in the number of imported cases, mainly from South and Southeast Asian countries [15–17]. Despite only sporadic abundance of *Aedes aegypti*, the species *Aedes albopictus* is widespread across Honshu Island and all western parts of Japan, theoretically allowing for chains of dengue transmission to exist [18–23]. Dengue was eliminated in the country by 1945 and transmission has not been observed for 70 years [24]; however, a German traveler visiting Japan in the summer of 2013 was later diagnosed with DENV2 infection upon returning to Germany [25]. In 2014, autochthonous transmission was confirmed in metropolitan Tokyo [26–31], resulting in a large outbreak involving a total of 160 confirmed cases, a shockingly high incidence for a previously dengue-free nation. It is now recognized that Japan is indeed at risk of dengue outbreaks during the summer season, indicating that a certain risk exists for the summer Olympic Games in 2020. This threat requires concrete planning for possible countermeasures in the event of another outbreak.

The 2014 dengue outbreak in central Tokyo was caused by a single serotype, DENV1, which showed high homology with the predominant circulating serotype in Southeast Asia [26]. There were two notable characteristics of this outbreak. First, of the total 160 people with a confirmed dengue diagnosis, 129 had visited or worked near Yoyogi Park, a national park that belongs to Shibuya Ward. Shibuya is a special ward that is a major commercial and business center. Shibuya has one of the busiest railway stations in the city, Shibuya Station, about 1 km from the park. Dengue transmission was concentrated in the park, where relatively high vector competence (mean biting rate 7.1 bites per person per 8 minutes) was observed [32]. Second, a few local residents seemed to remain for extended periods in Yoyogi Park (i.e., perhaps homeless people), and these people appeared to have been infected at a higher frequency than other individuals [33]; however, the role of these individuals in amplifying transmission as primary cases has not been verified. Once the outbreak was recognized in late August 2014, concerted efforts were made to contain spread of the virus, including mosquito control targeting both adults and larvae, disseminating news of the outbreak via mass media, communication of dengue risk by experts to raise public awareness, and even a total ban on entering the park. Descriptive documents with details of the outbreak are available but are mostly limited to Japanese language [26,28,30,33,34]. However, the effectiveness of interventions during the outbreak remains an important epidemiological question.

Mathematical modeling techniques are powerful tools for retrospective assessment of disease outbreaks. These methods include objective measurement of transmission such as the effective reproduction number, i.e., the actual average number of secondary cases generated by a single primary case, sometimes in the presence of interventions [35,36]. In the present study, we formulated a mathematical model and derived a likelihood function, aiming to estimate the effectiveness of interventions during the 2014 outbreak. Because several countermeasures were implemented on different dates during the outbreak, we calculated the effective reproduction numbers to assess the effectiveness of these interventions.

## Methods

### Epidemiological data

In Japan, DENV infection is categorized as a category IV disease, according to the Infectious Disease Law; thus, all physicians are required to notify diagnosed cases to the government via local health centers upon diagnosis [37]. The clinical characteristics of infection include (i) high-grade fever, which is typically biphasic, following an incubation period of 2–14 days [38,39]; (ii) headache, reddish face and/or conjunctivitis; (iii) general fatigue; (iv) muscle and joint pain, followed by (v) generalized rash that starts on the chest and abdomen. For patients with these characteristics, a physician must confirm the diagnosis via virus isolation, PCR method, detection of nonstructural protein 1, elevated IgM antibodies against DENV, or plaque reduction neutralization testing.

**Fig 1:**
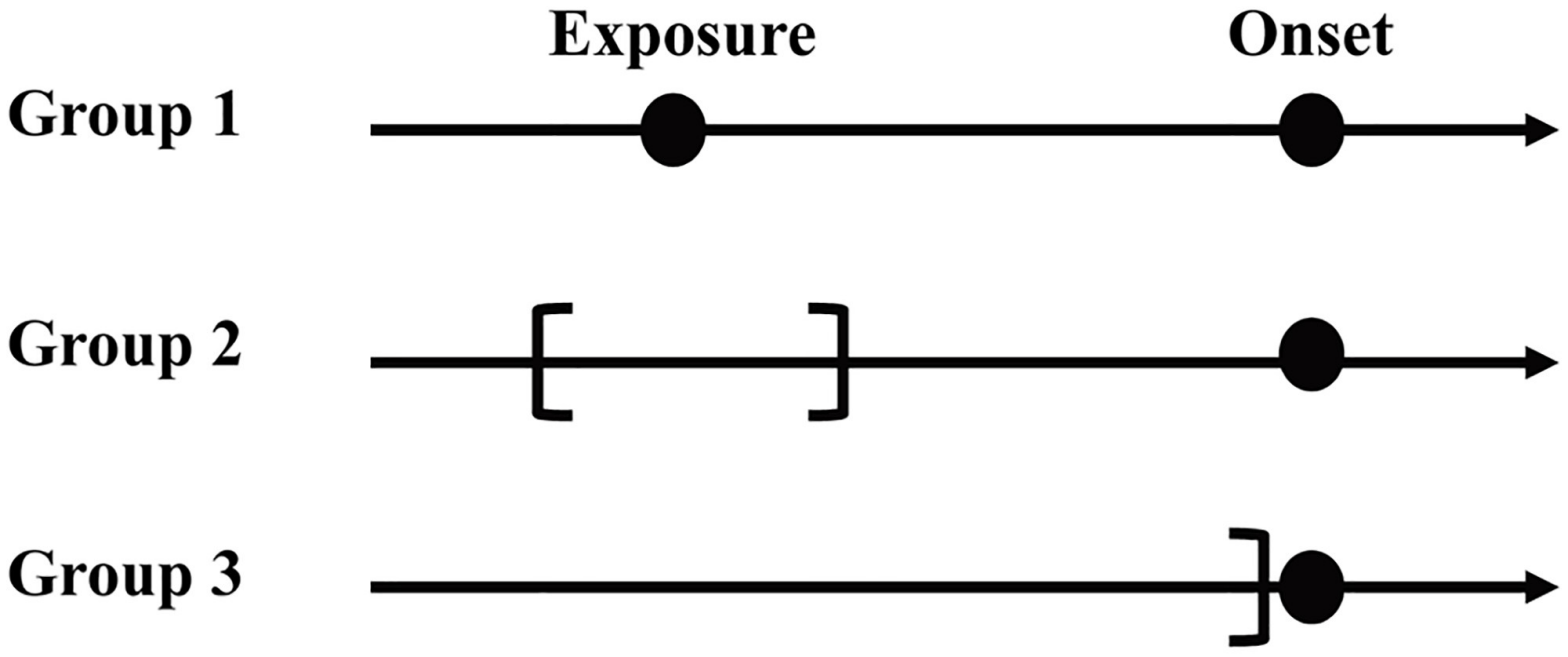
Illustrated time of exposure and classification of cases. In the present study, diagnosed cases of dengue virus fell into one of three groups. In Group 1, the exact date of exposure was known. In Group 2, exposure dates were interval censored, calling for interval-censored likelihood. In Group 3, no information was available with respect to time of exposure. Depending on this grouping, we used slightly different likelihood functions.

During the 2014 outbreak, notifications as well as details of the outbreak and interventions were summarized in an official report by the Tokyo metropolitan government [34]. In the present study, we retrieved the dates of exposure and illness onset from this report. The date of exposure was calculable because many cases were associated with exposure at Yoyogi Park. Individuals who did not have a history of visiting Yoyogi Park had a history of being bitten by mosquitoes in one of several other parks in the nearby Kanto region. However, the date of exposure was partly censored. Of the total 160 reported cases, four were excluded owing to the absence of information about illness onset (i.e., people who were serologically diagnosed, including local residents of the park). The remaining reported cases were statistically categorized into one of the following three groups (Fig 1):

1. cases exposed to DENV on a single day with a known date of illness onset (i.e., complete observation; Group 1, *n* = 79),
2. cases exposed to DENV for a certain number of days with a known date of illness onset (i.e., interval-censored observation of exposure; Group 2, *n* = 47),
3. cases without information of the date of exposure but with a known date of illness onset (i.e., missing observation of exposure; Group 3, *n* = 30).

Individuals in Group 2 visited Yoyogi Park on two or more consecutive days. Accordingly, the reported cases were categorized into Groups 1, 2, and 3 (S1 Fig).

In this outbreak, the actual primary case, which must be an imported case, was not identified. We defined the initial calendar day as *t*_0_, i.e., day 0 of the epidemic, which was not empirically observed. We assumed that exposure among secondary and subsequent generations of human cases began to occur after *t*_0_. The earliest observed date of exposure was 4 August 2014, and we denoted *d*_0_ as the gap number of days between *t*_0_ and 4 August (i.e., *d*_0_ = 4 August minus *t*_0_).

Table 1 shows the timeline of the outbreak. The index case, i.e., the first identified clinical case, had illness onset on 9 August and the confirmatory diagnosis was made on 26 August. Mosquito control and public notification of the outbreak started on 28 August; because infected adult *Aedes* mosquitos continued to be detected in Yoyogi Park, the government decided to close the park on 4 September.

**Table 1:** Event log of the 2014 dengue outbreak in Tokyo, Japan.

Date	Events
9 August 2014	Illness onset in the index case (with the earliest date of illness onset)
25 August 2014	Medical attendance of index case (10-year-old girl)
26 August 2014	Confirmed diagnosis of the index case and confirmation of the autochthonous transmission in Tokyo
†28 August 2014	Interventions against adult and larva of *Aedes albopictus* started in Yoyogi park along with public dissemination of the outbreak as a news
†4 September 2014	Ban on entrance to Yoyogi park
25 September 2014	Absence of virus detection in *Aedes albopictus* in Yoyogi park
7 October 2014	Illness onset in the last case

^†^ Key dates on which interventions were initiated (*T*_1_ and *T*_2_, respectively, in the main text).

### Epidemic curve as the probability distribution

Here, we describe the transmission dynamics of DENV in Tokyo using a mathematical model. First, we decomposed the generation time of DENV infection into two parts, i.e., (i) the time from illness onset in an infected human to secondary transmission in another human via a mosquito, denoted as the random variable, *t*_*Trans*_, and (ii) the time from infection in a human to their illness onset, again denoted by the random variable, *t*_*lP*_, corresponding to the intrinsic incubation period. Let *w*_t_ and *f*_s_ be the probability mass functions (pmf) to which random variables *t*_*Trans*_ and *t*_*lP*_ follow, respectively, and we assumed that both functions would be derived from the cumulative distribution functions of gamma distribution, *G*(*s*), i.e., *f*_s_ = *G*(*s*; *μ*_*lP*_, *σ*_*lP*_)−*G*(*s*−1; *μ*_*lP*_, *σ*_*lP*_) for *s*>0. The parameters *μ*_*lP*_ and *σ*_*lP*_ are the mean and standard deviation of the (intrinsic) incubation period in humans. Similarly, we assume that the parameters *μ*_Trans_ and *σ*_Trans_ would determine the mean and standard deviation of the pmf *w*_t_. Then, the pmf of the generation time, *g*_t_, defined as the time from infection in a human to infection in its secondary human case via a mosquito, can be modeled by convolution,

$$g_{t}=\sum_{\tau =0}^{t}f_{t-\tau }w_{\tau }.$$ (1)

As we did not know the parameters that govern *f*_s_ and *w*_t_, we jointly estimated them using other epidemiological parameters (see below). The abovementioned model does not explicitly account for the lifespan of the female *Aedes* species, which is considered to be 6 weeks or longer [40]. For simplicity, we ignored this matter, because the time scope of the Tokyo epidemic in 2014 was from 9 August to 7 October 2014, consistent with the average lifespan; thus, the empirically estimated generation time was sufficiently shorter than the lifespan.

Using *g*_t_, we devised a generation-dependent epidemiological model, which has been described elsewhere [41]; see S1 Text for the derivation of the generation-dependent model. In this model, we assumed that the epidemiological dynamics described by the generation-dependent model are what is expected in the absence of interventions. We defined the unobserved index case as generation 0. The index case produces generation 1, and the size of generation 1 is *R*_0_ cases with the relative timing of infection following *g*_t_ (i.e., following the infection time of the index case, there would be *R*_0_ *g*_t_ cases on day *t*). Subsequently, generation 1 produces *R*_1_ cases of generation 2, where *R*_1_ is the reproduction number of generation 1, and there would be *R*_0_*R*_1_ (*g* * *g*)_*t*_ cases as a function of time since index case *t*, where * is the convolution operator. If there are only two generations (excluding generation zero), the expected value of the incidence at *t* days since infection in the index case is *R*_0_*g*_*t*_ + *R*_0_*R*_1_ (*g* * *g*)_*t*_. Continuing this procedure through generation 4, and normalizing the quantity by the cumulative number of cases, we obtain the probability density function of infection, *h*(*t*), as

$$h(t)=\frac{R_{0}(g_{t}+R_{1}{\left(g\;*\;g\right)}_{t}+R_{2}R_{1}{\left(g\;*\;g\;*\;g\right)}_{t}+R_{3}R_{2}R_{1}{\left(g\;*\;g\;*\;g\;*\;g\right)}_{t})}{R_{0}+R_{1}R_{0}+R_{2}R_{1}R_{0}+R_{3}R_{2}R_{1}R_{0}},$$ (2)

where *R*_m-1_ denotes the reproduction number of generation *m*, describing the average number of secondary cases in generation *m* produced by a single primary case in generation (*m*−1), in the absence of interventions. *R*_0_ + *R*_1_*R*_0_ + *R*_2_*R*_1_*R*_0_ + *R*_3_*R*_2_*R*_1_*R*_0_ represents the total number of cases, considering up to the fourth generation. *R*_0_ is cancelled out and we have

$$h(t)=\frac{g_{t}+R_{1}{\left(g\;*\;g\right)}_{t}+R_{2}R_{1}{\left(g\;*\;g\;*\;g\right)}_{t}+R_{3}R_{2}R_{1}{\left(g\;*\;g\;*\;g\;*\;g\right)}_{t}}{1+R_{1}+R_{2}R_{1}+R_{3}R_{2}R_{1}}.$$ (3)

Eq (3) describes the epidemic curve of infection as the probability distribution in the absence of interventions. It should be noted that *h*(*t*) is a function of the time of infection, not illness onset.

Though only arithmetically, *R*_0_ can be calculated by dividing the observed cumulative number of cases by (1 + *R*_1_ + *R*_2_*R*_1_ +*R*_3_*R*_2_*R*_1_). Additionally, we incorporated the effectiveness of the interventions implemented during the 2014 outbreak. As Table 1 shows, mosquito control started on 28 August (*T*_1_); subsequently, Yoyogi Park was closed on 4 September (*T*_2_). We wished to assess the effectiveness of these two interventions separately. We used relative reduction in the reproduction number, *ε*(*t*), defined as follows:

$$\varepsilon (t)=\left\{ \begin{array}{cc} 1 & t<T_{1}\\ \varepsilon_{1} & T_{1}\leq t<T_{2}\\ \varepsilon_{1}\varepsilon_{2} & T_{2}\leq t. \end{array}.\right.$$ (4)

Using *h*(*t*)*ε*(*t*), we described the observed epidemic dynamics. Normalizing the product, *h*(*t*)*ε*(*t*), we obtained the probability density of the epidemic curve of infection,

$$u(t)=\frac{h(t)\varepsilon (t)}{\sum_{\tau =0\;}^{T}h(\tau )\varepsilon (\tau )},$$ (5)

where *T* is the last date of the outbreak. Using the parameterized incidence function, *u*(*t*), the effective (or instantaneous) reproduction number *R*(*t*) is calculated as the estimator of the renewal equation [42,43], i.e.,

$$R(t)=\frac{u_{t}}{\sum_{\tau =0}^{t}u_{t-\tau }g_{\tau }}.$$ (6)

Note that *u*(*t*) is now described on a daily basis; thus, *u*_t_ represents the daily probability on day *t*.

### Likelihood function

We did not know the first day of exposure, *t*_0_; therefore, we varied *d*_0_ within a plausible range, using 3 to 9 days as a theoretically possible range [34]. Assuming that the incubation period was independently and identically distributed, our mathematical model was formulated using the convolution of infection probability, *u*_t_, and distribution of the incubation period, *f*_s_. We let *t*^e^ be the date of exposure and *t*^s^ be the date of illness onset. Unknown parameters ***θn*** = {*μ*_*lP*_, *σ*_*lP*_, *μ*_*Trans*_, *σ*_*Trans*_, *R*_1_, *R*_2_, …, *R*_*n*−1_, *ε*_1_, *ε*_2_} were estimated using a maximum likelihood method. The exact number of generations was unknown; thus, we fit three different models with a variable number of generations (i.e., *n* = 2, 3, and 4 excluding generation 0) and later compared the Akaike information criterion values with a correction for small sample size (AICc) and mean squared error (MSE). We did not consider additional generations, because more generations unrealistically required shorter duration of the extrinsic incubation period (EIP). As a sensitivity analysis, we compared models with and without *ε*_1_ and *ε*_2_, i.e., models in which (i) two effectiveness measures are jointly estimated (i.e., *ε*_1_≠1 and *ε*_2_≠1), (ii) only the park closure effect is estimated (*ε*_1_ = 1 and *ε*_2_≠1), (iii) only mosquito control and public awareness campaigns are factored in (*ε*_1_≠1 and *ε*_2_ = 1), and (iv) no effect of control measures are taken into account (*ε*_1_ = 1 and *ε*_2_ = 1). To this end, we fixed the generation time distribution, using parameters informed from the best model with *ε*_1_≠1 and *ε*_2_≠1 and jointly estimated only *R*_i_ and these effectiveness parameters, comparing AICc values across different models.

For case *i ∈* Group 1where the case has exact dates of exposure $t_{i}^{e}$ and symptom onset $t_{i}^{s}$ [44], the likelihood of observation is

$$L_{i}^{1}(\theta_{n};t_{i}^{e},t_{i}^{s},d_{0})=u_{z(t_{i}^{e},d_{0})}f_{t_{i}^{s}-t_{i}^{e},}$$ (7)

where z(τ, *d*_0_) = *τ* − t* + *d*_0_ + 1 and t* represents the first exposure time (4 Aug 2014). For case *j* in Group 2, the case has an interval-censored observation for exposure date $t_{j}^{e}$, ranging from the first date of visit $E_{j}^{L}$ to the last date of visit $E_{j}^{R}$ (i.e., $t_{j}^{e}\in (E_{j}^{L},E_{j}^{R}]$) [44,45]. The likelihood function for case *j* in Group 2 is

$$L_{j}^{2}(\theta_{n};E_{j}^{L},E_{j}^{R},t_{j}^{s},d_{0})=\sum_{{\tau =E}_{j}^{L}}^{E_{j}^{R}}u_{z(\tau ,d_{0})}f_{t_{j}^{s}-\tau .}$$ (8)

For case *k* in Group 3, the exposure can take place from time 0 to the onset of symptoms (i.e., $t_{k}^{e}\in [t_{0},t_{k}^{s}]$),

$$L_{k}^{3}(\theta_{n};t_{k}^{s},d_{0})=\sum_{\tau =t_{0}}^{t_{k}^{s}}u_{z(\tau ,d_{0})}f_{t_{k}^{s}-\tau .}$$ (9)

In addition, cases in Group 1 were used to estimate the incubation period, i.e.,

$$L^{IP}(\mu_{IP},\;\sigma_{IP};t^{e},t^{s})=\prod_{i=1}^{n_{1}}f_{t_{i}^{s}-t_{i}^{e},}$$ (10)

where *n*_*m*_ is the total number of cases in Group *m* = {1, 2, 3}. In Eq (10), we did not consider interval-censored data because these are already reflected in Eq (8) with exposure distribution *u*(*t*) during the interval. The total likelihood function is

$$L(\theta_{n};t^{e},t^{s},E^{L},E^{R},d_{0})=\prod_{i=1}^{n_{1}}L_{i}^{1}\prod_{j=1}^{n_{2}}L_{j}^{2}\prod_{k=1}^{n_{3}}L_{k}^{3}\prod_{i=1}^{n_{1}}L_{i}^{IP}.$$ (11)

For the estimation of unknown parameters **θ**_n_, we used the method of maximum likelihood estimation to minimize the negative likelihood L(**θ**_n_; *t*^*e*^, *t*^*s*^, *E*^*L*^, *E*^*R*^, *d*_0_). We did not impose any constraints for the range of parameters.

The 95% confidence interval (CI) of the effective reproduction number was computed with a parametric bootstrap method. We let *H*(θ*) be the Hessian matrix for estimated values θ*. The 100 sets of parametric bootstrap samples were generated from the multivariate normal distribution with the mean and covariance, the latter of which was obtained with *diag*(*H*^-1^(θ*)). Simulating 100 times, 2.5th and 97.5th percentile values of the resampled distribution were used to calculate the 95% CI. All statistical analyses were conducted using R 3.5.1 (R Development Core Team [46]).

### Ethical considerations

In the present study, we analyzed data that are publicly available [34]. As such, the datasets used in this study were de-identified and fully anonymized in advance; the analysis of publicly available data with no identifying information does not require ethical approval.

### Data sharing policy

Individual datasets for the date of exposure and illness onset are available in S1 Table; these also served as the data source of the epidemic. The R programming code for the two-generation model has been made publicly available at https://github.com/Biomath-2019/Dengue.

## Results

**Fig 2:**
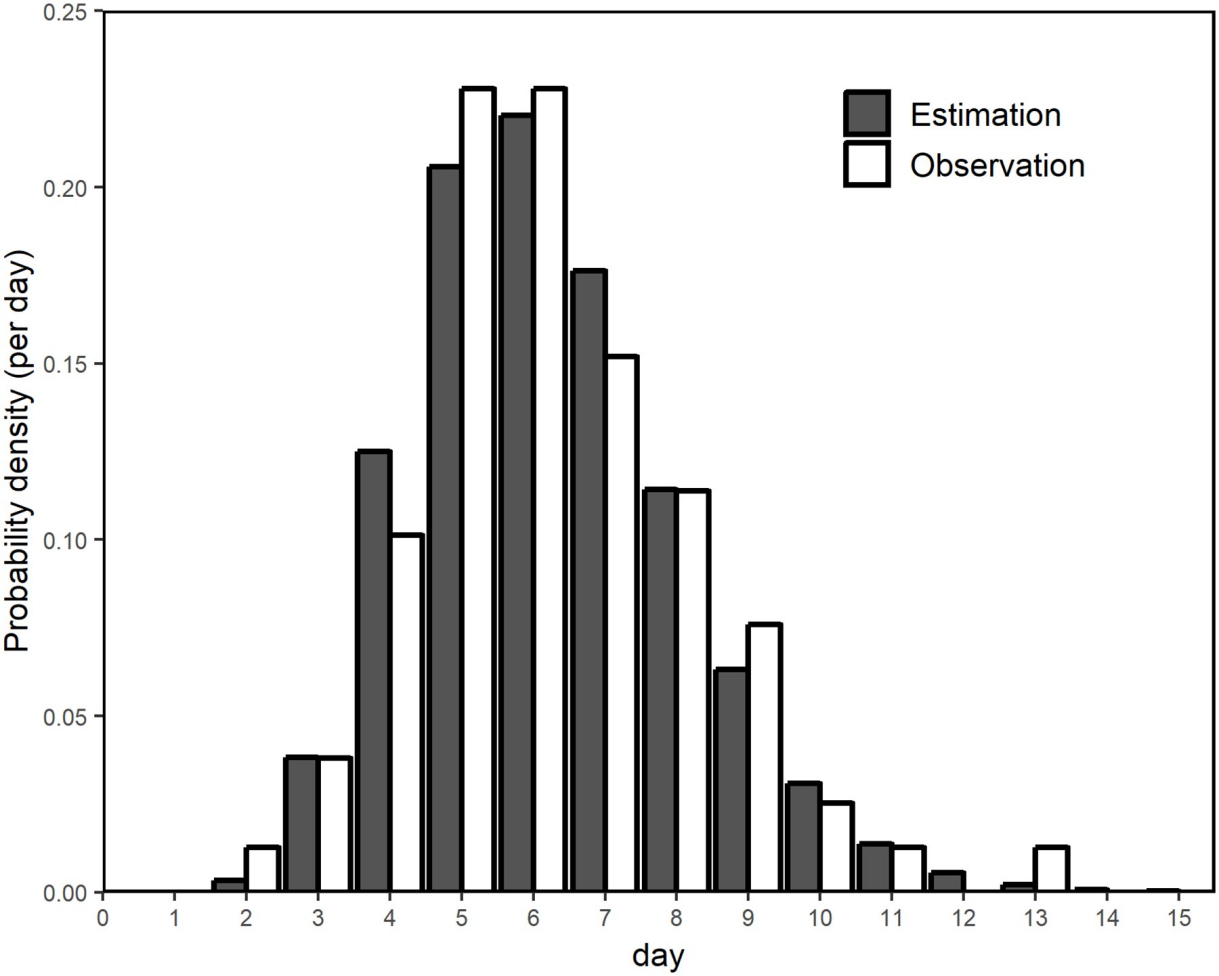
Comparison between observed and estimated distributions of the incubation period. Horizontal axis represents the incubation period of dengue virus infection, i.e., the time from mosquito exposure to illness onset. Grey bars represent observed frequency of the incubation period; white bars represent the estimated probability distribution.

Fig 2 shows the incubation period distribution, confirming that the observed and estimated frequencies agreed well. The mean and standard deviation of the incubation period were estimated at 5.8 (95% CI: 5.5, 6.0) days and 1.8 (95% CI: 1.6, 2.1) days, respectively.

**Fig 3:**
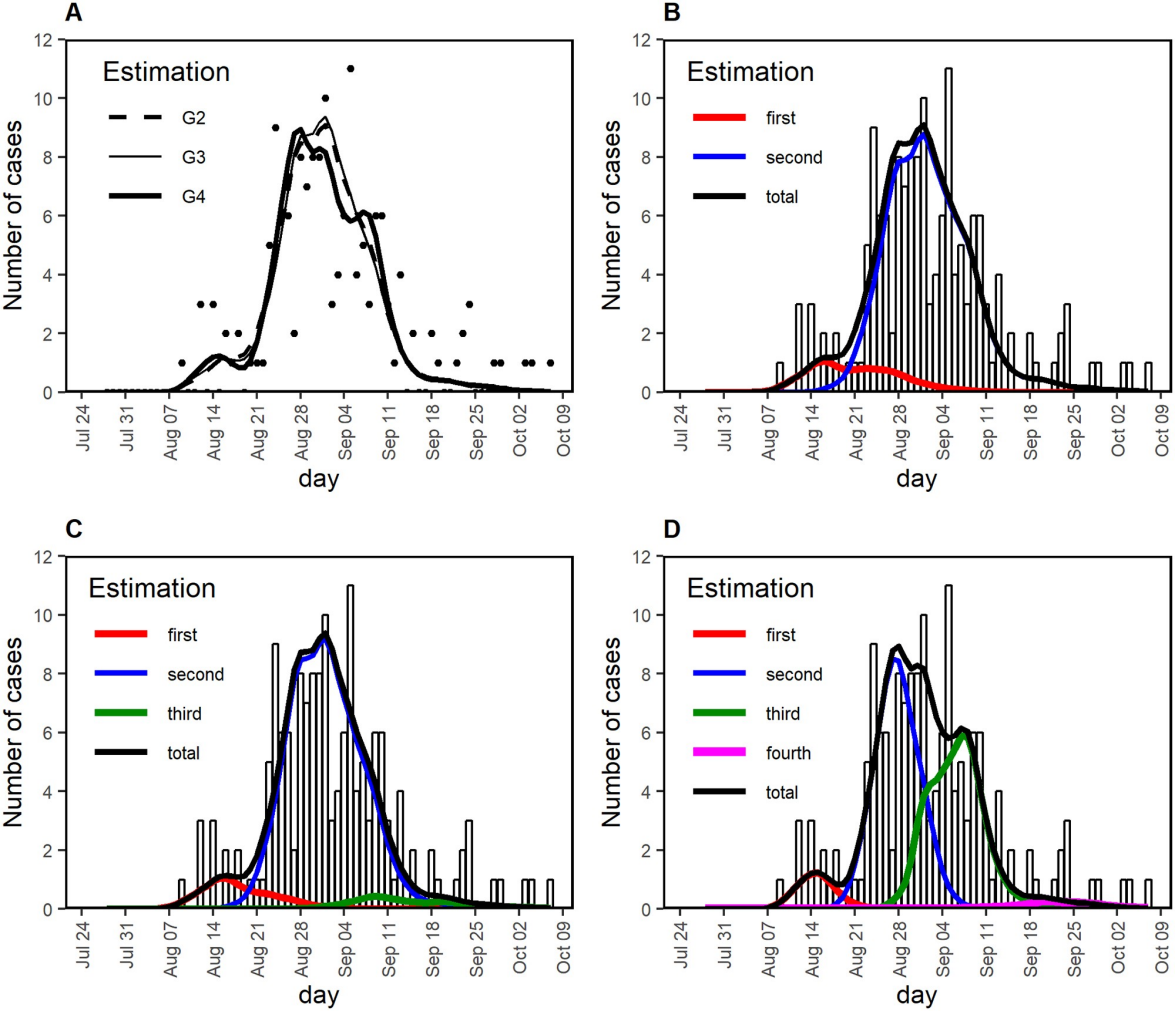
Comparison between observed and estimated epidemic curves of the 2014 dengue outbreak in Tokyo. (A) Total number of cases with time of illness onset. Black dots show the observed number of cases; lines are derived from mathematical models with different numbers of assumed generations of infection, G2, G3, and G4, representing a total number of generations of 2, 3, and 4, excluding generation zero. (B–D) Comparison between observed and estimated cases by different numbers of assumed generations of infection, G2, G3, and G4, corresponding to panels B, C, and D, respectively. The observed number of cases is shown as bars. Red, blue, green, and magenta lines depict the estimated cases generated by first, second, third, fourth generation cases, respectively. Black line represents the estimated total number of cases.

Fitting three different models with a different assumed number of generations, the observed temporal patterns were well captured overall (Fig 3A). Identifying a few best-fit models with a different number of generations, the most likely first date of exposure was in the range 26–28 July 2014, dating back 7–9 days from 4 August 2014 (S2 Table). Regardless of the assumed number of generations, the first generation (i.e., generation 1) coincided with a small peak in the epidemic curve on 14 August; generation 2 was considered to be primarily responsible for the highest peak at the end of August (Fig 3B–3D). Only when generation 4 was assumed to have existed, generation 3 was considered responsible for a small peak on about 7 September 2014.

Table 2 summarizes the parameter estimates derived from the identified best model, given the assumed number of generations. The mean generation time from DENV infection in a human to another DENV infection in a human via a mosquito bite (i.e., *μ*_IP_ + *μ*_Trans_) was estimated at 17.2 days, 16.1 days, and 12.4 days for the assumed number of generations 2, 3, and 4, respectively. As the assumed number of generations increased, the estimated mean and standard deviation became significantly smaller, reflecting that the explainable transmission dynamics greatly varied by the assumed number of generations. The reproduction numbers in the absence of interventions were greater than the value of 1 by generation 2 (i.e., *R*_1_) for the three models, and by generation 3 (i.e., *R*_2_) if the assumed number of generations was 4. It should be noted that if the number of generations was 2, then the reproduction number of generation 3 (i.e., *R*_2_) was not estimated but must have been zero (owing to the absence of generation 3). It should be noted that the latest estimate of *R*_i_ was below the value of 1 and this was not surprising, because the epidemic came to an end in October 2014.

**Table 2:** Estimates of the best models by number of generations.

Parameters	Two-generation model (G2)	Three-generation model (G3)	Four-generation model (G4)
*d*_0_	7	9	8
*μ*_IP_	5.8 (5.5, 6.0)	5.8 (5.5, 6.0)	5.8 (5.5, 6.1)
*σ*_IP_	1.8 (1.6, 2.1)	1.8 (1.6, 2.1)	1.9 (1.6, 2.1)
*μ*_Trans_	11.4 (9.1, 14.3)	10.3 (8.4, 12.6)	6.6 (5.8, 7.5)
*σ*_Trans_	7.3 (5.5, 9.6)	4.9 (3.3, 7.2)	2.0 (1.2, 3.4)
*R*_0_	15.1	13.6	4.0
*R*_1_	9.3 (4.7, 18.6)	8.8 (4.5, 17.1)	5.4 (2.7, 10.5)
*R*_2_	-	0.2 (0.1, 0.8)	4.3 (1.1, 16.8)
*R*_3_	-	-	0.4 (0.2, 1.0)
*ε*_1_	0.7 (0.3, 0.9)	0.7 (0.2, 1.0)	0.3 (0.1, 0.7)
*ε*_2_	0.6 (0.2, 0.9)	0.8 (0.1, 1)	0.4 (0.1, 0.7)
MSE	2.9	2.8	2.6
AICc	1855.0	1856.0	1855.2

Note: Two-generation, three-generation, and four-generation models indicated that there were a total two, three, and four generations, excluding generation zero. Mean *μ*_IP_ and standard deviation *σ*_IP_ of the incubation period were assumed as the parameters governing the gamma distribution. Mean *μ*_Trans_ and standard deviation *σ*_Trans_ of the duration of the generation times, other than incubation period, were also assumed to follow a gamma distribution. *R*_n-1_ is the reproduction number of the *n*^th^-generation infection in the absence of interventions. To account for the effectiveness of interventions, *ε*_i_ was factored into the model as the relative reduction in the reproduction number owing to mosquito control (*ε*_1_) and park closure (*ε*_2_). MSE represents the mean squared error. AICc is the Akaike information criterion with a correction for small sample size, calculated as AICc = AIC+ (2*k*(*k*+1))/(*n*-*k*-1), where *n* is the number of data points (i.e., n = 156) and AIC = 2*NLL*+2*k* where *NLL* is the negative log-likelihood and *k* is the number of parameters.

The model average is the average of three former modes with weight as a function of AIC, which was calculated as 0.35, 0.25, and 0.40.

**Fig 4:**
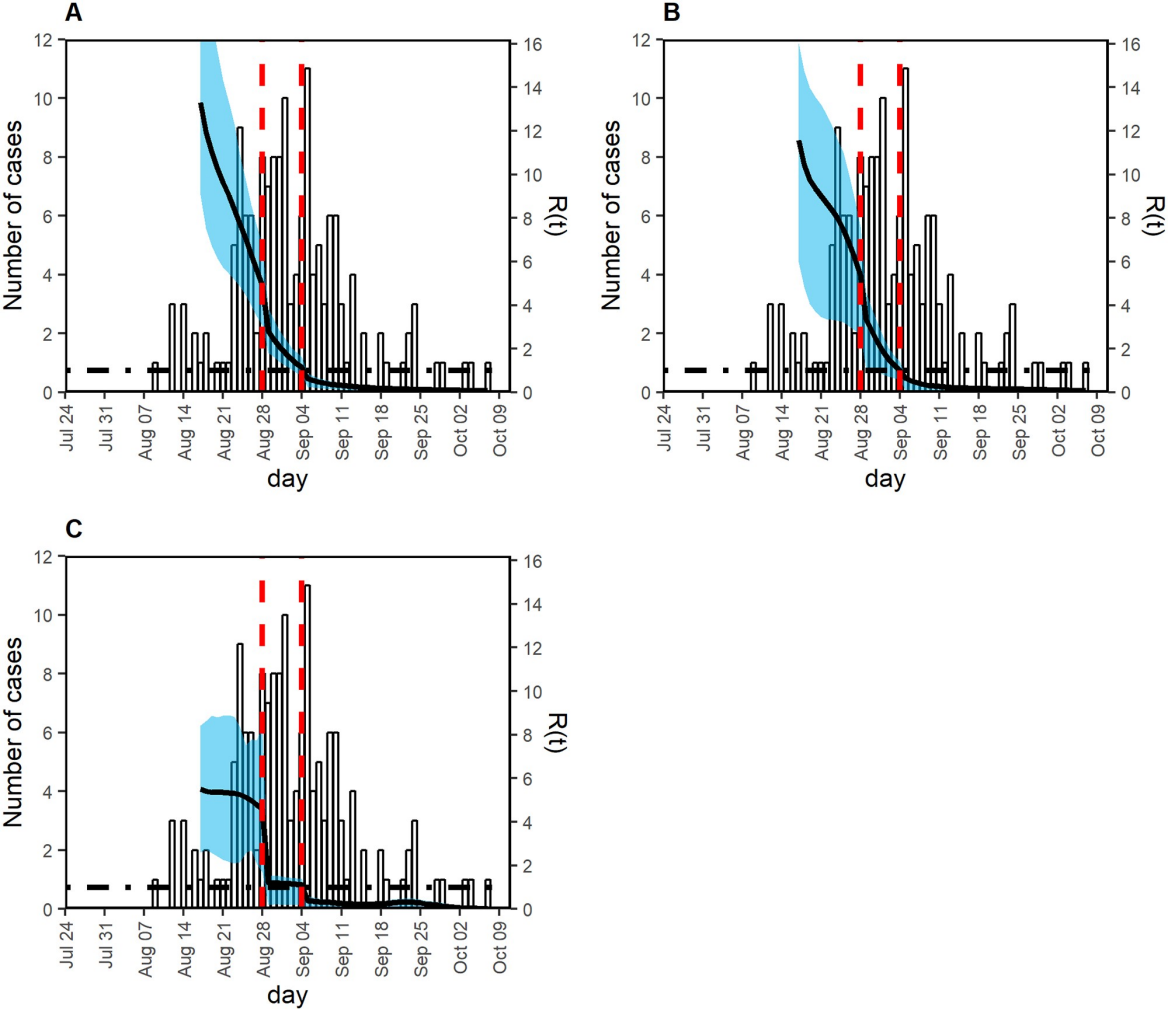
Effective reproduction number in three models with a different number of generations. Left vertical axis shows the observed number of cases (i.e., incidence as a function of the date of illness onset), and the right vertical axis shows the effective reproduction number, illustrated using a black solid line. Two red vertical dashed lines indicate times at which interventions started. The earlier one (from 28 August) included mosquito control and dissemination of outbreak information via mass media. The later vertical line indicates the date on which Yoyogi Park was closed. Black dot-dashed horizontal line indicates the threshold value 1 for the effective reproduction number, below which the outbreak will eventually be controlled. The effective reproduction number was estimated, assuming three different numbers of generations of infection, i.e., (A) two, (B) three, and (C) four generations excluding generation zero. Owing to the uncertainty of estimation during the very early stage of the epidemic, the effective reproduction number was plotted from 17 August. The shaded cyan area represents the 95% confidence interval (CI) of the effective reproduction number, calculated using 100 bootstrap samples.

The mosquito control and public awareness campaigns from 28 August 2014 were considered to have definitely reduced transmission, with secondary transmission reduced by an estimated 30%–70%, with a wide confidence interval (Table 2). Using the AIC value as the weight, the ensemble estimates of *ε*_1_ and *ε*_2_ following model averaging over different assumed numbers of generations were 0.6 and 0.6, respectively. Despite these reductions, it appeared that the estimated effective reproduction number did not decline below the threshold value of 1 (Fig 4). However, park closure in combination with the mosquito control and awareness campaigns successfully reduced the reproduction number. The relative reduction in the effective reproduction number owing to the closure of Yoyogi Park was estimated to be 20%–60%, again with a wide confidence interval (Table 2); however, the combined effect was estimated to be as large as a 44%–88% reduction in the reproduction number. These findings were robust for the assumed number of generations.

We conducted model comparisons to assess the importance of accounting for the effectiveness of the abovementioned control measures. The sensitivity results are summarized in S3 Table. It appeared that the latest estimate of the reproduction number was sensitive to the presence of effectiveness parameters (ε_1_ and ε_2_). Regardless of whether the assumed number of generations was 2, 3, or 4, AICc values of the model with both ε_1_ and ε_2_ were minimal (AICc = 1855.1, 1856.0, and 1855.2 for models with 2, 3, and 4 generations, respectively), outperforming models without one or both effectiveness parameters. S2 Fig compares the reproduction number for two-generation model over time, hypothetically examining counterfactual scenarios in which the relative reduction in the reproduction number during park closure, *ε*_2_ (and mosquito control, measured by *ε*_1_) is assumed at 1. If *ε*_2_ was 1, the time at which the reproduction number declines would be delayed, indicating that the park closure has played an important role in reducing the transmissibility.

## Discussion

In the present study, we performed a retrospective epidemiological assessment of interventions to control the 2014 dengue outbreak in Tokyo, using a limited number of confirmed cases (*n* = 160). Because the anticipated number of generations of infection was limited, and also because we sought to conduct careful evaluation of the causal association between the timing of interventions and the effective reproduction number *R*_t_, we did not use the structured compartmental modeling approach (e.g., using ordinary differential equations). Instead, we developed a novel method to directly parameterize the incidence of infection, by convoluting the incidence of infection with the incubation period, allowing us to precisely incorporate the timing of interventions and observe their effect on virus transmission dynamics on a given day. The proposed method suitable for application (i) when the generation structure is imaginable from a published estimate of the mean generation time or visually identifiable from the observed epidemic curve, (ii) when the time of infection needs to be modeled in relation to the timing of interventions, and (iii) when the observed data include doubly interval-censored data. As a consequence of our analyses, the effectiveness of mosquito control, dengue risk communication to elevate public awareness, and closure of the focal area of transmission were objectively evaluated in relation to the effective reproduction number of DENV infection.

In practical terms, what the present study adds to the literature is that in the case of the 2014 dengue outbreak in Tokyo, all control measures that we explored (i.e., mosquito control, public awareness campaigns, and park closure) acted as essential factors governing the observed patterns of the epidemic. This notion is supported by our model comparisons in which both *ε*_1_ and *ε*_2_ were required to act as free parameters, to better describe the observed epidemic dynamics. Of these interventions, mosquito control and raising public awareness were not sufficiently effective to break the chain of transmission, as they maintained *R*_t_>1, although the reproduction number exhibited a decreasing trend due to decrease in the observed incidence. Although mosquito control from 28 August 2014 was very intensive, DENV-positive *Aedes* were detected after these measures had been implemented [34]. To fully halt virus transmission, the combined effect of mosquito control, public awareness campaigns, and park closure was needed for a substantial reduction in *R*_t_; this joint reduction effect was estimated to be 44%–88%. Therefore, according to our model results, we can conclude that control of the dengue outbreak at local level in Tokyo was essential to describe the empirically observed data and successful in reducing transmissions. It should be noted that the park closure effect includes not only the prevention of exposure among susceptible visitors but also removal of infectious hosts, including local residents of the park, from the focal area of transmission.

In developing the generation-based modeling approach, the proposed system was quantified with estimated mean generation time from 12 to 17 days. Considering the published length of the EIP [38,39,47], with summer temperatures in metropolitan Tokyo above 30 °C in August, a mean generation time of about 2 weeks is regarded as a reasonable length or slightly shorter than the temperature-dependent estimate. Our estimate was consistent with an empirical estimate by Siraj et al. [48], indicating that a mean generation interval of 17 days occurs with the highest probability at 30 °C, although the estimate was obtained for *Aedes aegypti* and not for *Aedes albopictus*, the latter of which is abundant in Japan. The mean incubation period of 5.8 days is also consistent with the literature [37], and we attained a finer estimation than that of Ishikawa et al. [30]; those authors used only known 67 intrinsic incubation periods for the estimation (with an estimated mean of 6.3 days), potentially resulting in a biased estimate of the variance owing to small sample size.

Although we imposed three different assumptions, i.e., three different numbers of generations, the resulting AIC values were comparable, and we were unable to select the best model. However, the model with two generations alone indicated that the outbreak came to an end with the latest estimate of a generation-dependent reproduction number (of generation 1) as large as 9.3; if this model result were true, the reproduction number of the subsequent generation (generation 2) had to abruptly drop to 0. As mentioned in the Results, regardless of the assumed number of generations, the latest estimate of *R*_i_ was below the value of 1 because the epidemic came to an end in October 2014. However, using the model with two generations, this had to happen quite abruptly, with a drop from 9.3 to 0. Using the model with four generations, the mean generation time had to be 12 days, allowing only about 6 to 7 days from illness onset in an infected person to EIP in a mosquito to biting a susceptible person. Thus, there was some interplay between the assumed number of generations and the resulting estimates, i.e., as the number of generations increased, the generation time was estimated to be shorter. As long as we cannot specify the exact number of generations, the only approach to address relevant uncertainty is to use multiple models with different numbers of generations, to verify that our practical conclusions about the effectiveness of interventions would not change drastically by varying the number of generations.

The present study was not free from limitations. First, this study rested on confirmed dengue cases in patients with symptomatic illness who undertook testing. Febrile patients who had visited Yoyogi Park were advised to seek medical attention during the outbreak; however, a substantial number of asymptomatic infections would have been missed [49], and the present evaluation was made based only on diagnosed cases. Thus far, we have failed to explicitly estimate the ascertainment rate or number of asymptomatic infections using the observed empirical data. Second, Yoyogi Park was undoubtedly the focal area of transmission, but later transmission occurred in other parks in the Kanto region. In addition to spatial heterogeneity, the exposure behaviors in those parks were not rigorously traced, leading to substantial uncertainty in the empirical data (i.e., many cases belonging to Group 3 in our likelihood), thus making later epidemic data difficult to be captured by our simple model. Third, qualitatively, local residents of Yoyogi Park were suspected of being amplifiers of transmission. However, it was not possible to trace the behavior of these infected individuals, e.g., when they left Yoyogi Park and where they went after leaving the park, including whether they moved to another park as a next destination, thus being responsible for causing subsequent cases. It must be remembered that our high estimate of the reproduction number in the early stage of the epidemic could have reflected the existence of superspreaders. Fourth, we ignored environmental and ecological factors in our model [50–52]. The temperature mostly remained stable during the course of the 2014 outbreak; thus, the EIP can be assumed to be approximately stable. However, we ignored rainfall, which could have altered the population dynamics of *Aedes* species. Fifth, we examined only a limited number of assumed generations; theoretically, there could be six, seven, or an even greater number of generations explaining the observed epidemiological dynamics. However, considering the published EIP, it was implausible that there were six or more generations during the course of the 2014 outbreak.

Despite these limitations, we strongly believe that among the existing models, our model provides the most meticulous approach to account for the exact impact of the intervention start date in changing the dynamics of dengue infection over time, because we directly modeled the time of infection in relation to the timing of interventions while maximally using interval-censored data of exposure and illness onset among cases. We successfully captured that impact using convolution of the incidence and the incubation period. By combining mosquito control, public awareness campaigns, and park closure, dengue control during the 2014 outbreak in Tokyo was highly successful. Should a similar event happen in the future, concerted efforts including similarly combined interventions, accompanied by identification of the location of exposure, should be implemented.

## Supporting information

S1 Fig Temporal distribution of dengue by three classification groups of datasets. According to statistical information of the date of exposure, cases were classified into three groups: Group 1, exact date of exposure was known; Group 2, exposure dates were interval censored; and Group 3, no information was available. In the figure, the date of illness onset is shown for these three groups. Light grey, white, and dark grey bars represent cases in Groups 1, 2, and 3, respectively.
(TIFF)

S2 Fig Effective reproduction number with two-generation model. Left vertical axis shows the observed number of cases (i.e., incidence as a function of the date of illness onset), and the right vertical axis shows the effective reproduction number, illustrated using a black solid line. Two red vertical dashed lines indicate times at which interventions started. The earlier one (from 28 August) included mosquito control and dissemination of outbreak information via mass media. The later vertical line indicates the date on which Yoyogi Park was closed. Black dot-dashed horizontal line indicates the threshold value 1 for the effective reproduction number, below which the outbreak will eventually be controlled. The effective reproduction number was estimated, assuming two generations of infection and using the estimated effectiveness values *ε*_1_ and *ε*_2_. Blue line shows the best fit, while dashed orange shows when *ε*_2_ was artificially assumed as 1. Similarly, dashed light green line shows when both *ε*_1_ and *ε*_2_ were artificially assumed as 1.
(TIFF)

S1 Table Dates of exposure and illness onset among a total of 156 dengue fever cases in Tokyo, Japan, 2014. (DOCX)

S2 Table Comparison of model fit by the number of generations and time lag between infection in the unobserved index case and observed first exposure (4 August 2014). (DOCX)

S3 Table Comparison of model fit by the number of generations and four settings of hypothesized effectiveness measures. (DOCX)

S1 Text Derivation of the generation-dependent model. (DOCX)
