# Supplementary figures and images for: Assessing dengue control in Tokyo, 2014
Source: PLoS Negl Trop Dis. 2019 Jun 21;13(6):e0007468. doi: 10.1371/journal.pntd.0007468 (PMC6588210; doi:10.1371/journal.pntd.0007468)

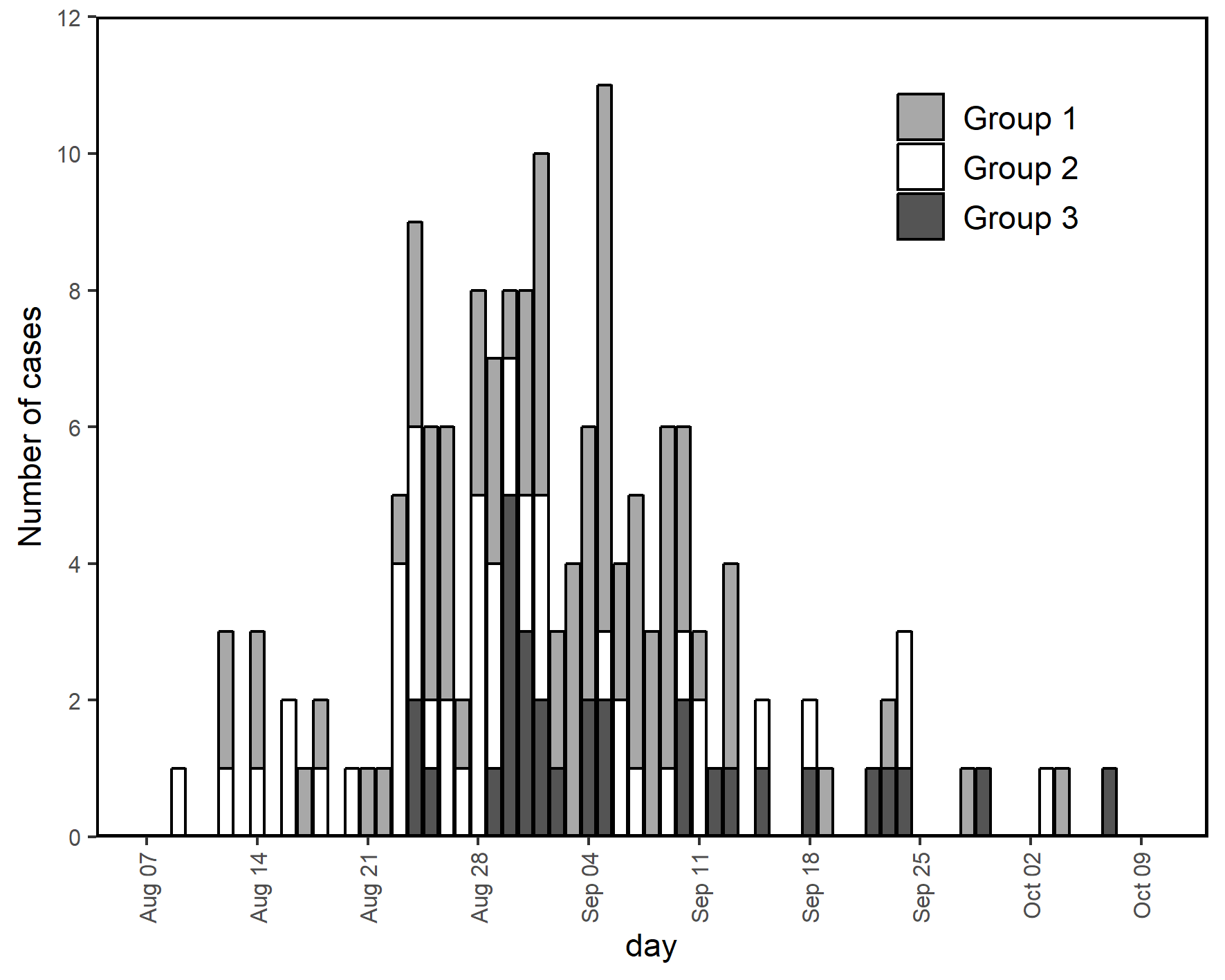

Supplement: S1 Fig — According to statistical information of the date of exposure, cases were classified into three groups: Group 1, exact date of exposure was known; Group 2, exposure dates were interval censored; and Group 3, no information was available. In the figure, the date of illness onset is shown for these three groups. Light grey, white, and dark grey bars represent cases in Groups 1, 2, and 3, respectively. (TIFF) [file pntd.0007468.s001.tiff]

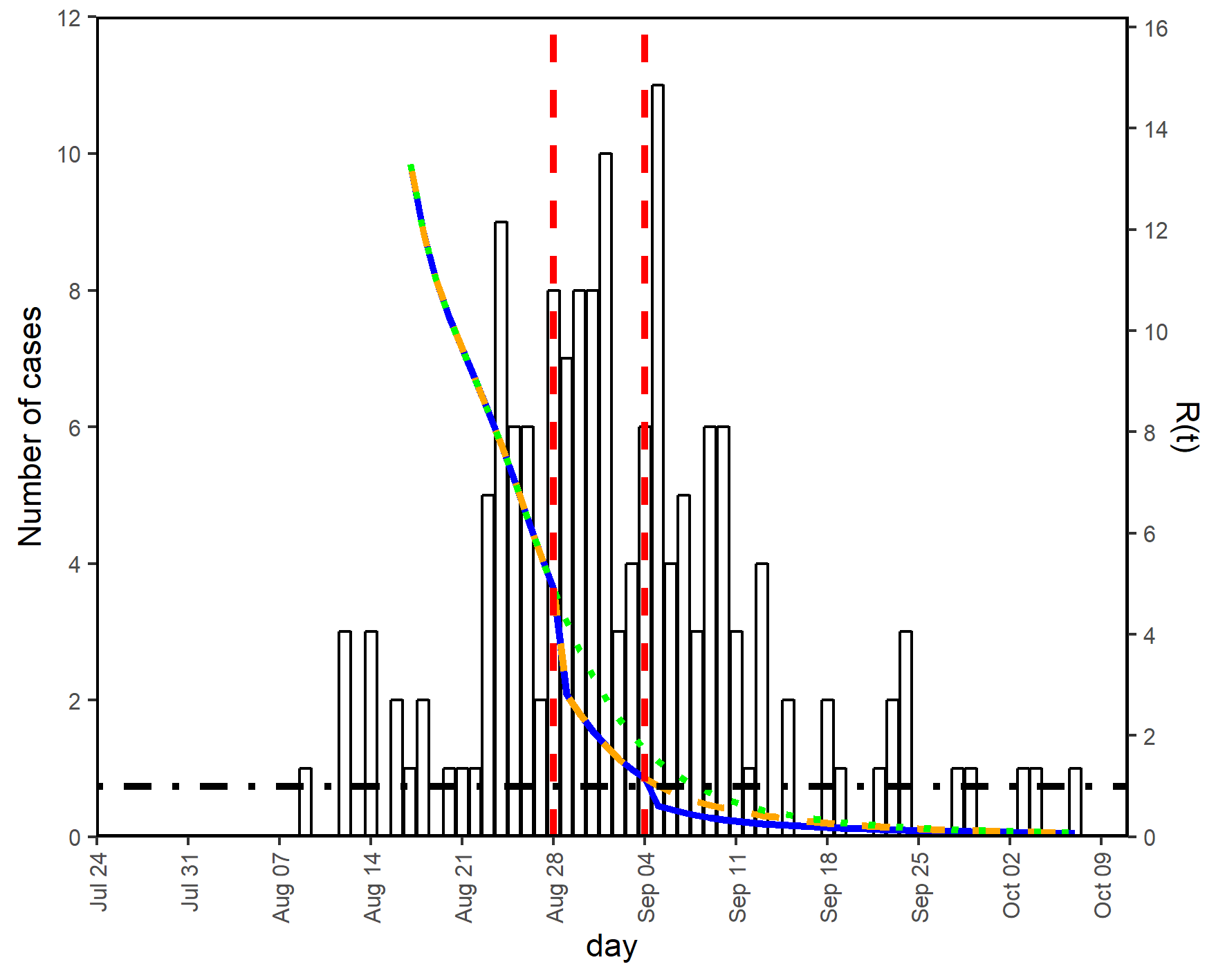

Supplement: S2 Fig — Left vertical axis shows the observed number of cases (i.e., incidence as a function of the date of illness onset), and the right vertical axis shows the effective reproduction number, illustrated using a black solid line. Two red vertical dashed lines indicate times at which interventions started. The earlier one (from 28 August) included mosquito control and dissemination of outbreak information via mass media. The later vertical line indicates the date on which Yoyogi Park was closed. Black dot-dashed horizontal line indicates the threshold value 1 for the effective reproduction number, below which the outbreak will eventually be controlled. The effective reproduction number was estimated, assuming two generations of infection and using the estimated effectiveness values ε1 and ε2. Blue line shows the best fit, while dashed orange shows when ε2 was artificially assumed as 1. Similarly, dashed light green line shows when both ε1 and ε2 were artificially assumed as 1. (TIFF) [file pntd.0007468.s002.tiff]
