# Supplementary material for: Assessing dengue control in Tokyo, 2014
Source: PLoS Negl Trop Dis. 2019 Jun 21;13(6):e0007468. doi: 10.1371/journal.pntd.0007468 (PMC6588210; doi:10.1371/journal.pntd.0007468)
Supplement: S1 Table — (DOCX) [file pntd.0007468.s003.docx]

## S1 Table. Dates of exposure and illness onset among a total of 156 dengue fever cases in Tokyo, Japan, 2014

| **Time of illness onset** | **Exposure time** | **Group** | **Case ID [34]** |
| --- | --- | --- | --- |
| 12 Aug | 4 Aug | 1 | 52 |
| 12 Aug | 7 Aug | 1 | 78 |
| 14 Aug | 10 Aug | 1 | 25 |
| 14 Aug | 9 Aug | 1 | 50 |
| 17 Aug | 10 Aug | 1 | 10 |
| 18 Aug | 11 Aug | 1 | 12 |
| 21 Aug | 16 Aug | 1 | 11 |
| 22 Aug | 17 Aug | 1 | 15 |
| 23 Aug | 10 Aug | 1 | 13 |
| 24 Aug | 20 Aug | 1 | 7 |
| 24 Aug | 17 Aug | 1 | 18 |
| 24 Aug | 20 Aug | 1 | 22 |
| 25 Aug | 20 Aug | 1 | 17 |
| 25 Aug | 18 Aug | 1 | 21 |
| 25 Aug | 18 Aug | 1 | 29 |
| 25 Aug | 19 Aug | 1 | 30 |
| 26 Aug | 21 Aug | 1 | 23 |
| 26 Aug | 19 Aug | 1 | 38 |
| 26 Aug | 21 Aug | 1 | 39 |
| 26 Aug | 22 Aug | 1 | 57 |
| 27 Aug | 25 Aug | 1 | 59 |
| 28 Aug | 20 Aug | 1 | 14 |
| 28 Aug | 22 Aug | 1 | 51 |
| 28 Aug | 23 Aug | 1 | 77 |
| 29 Aug | 22 Aug | 1 | 47 |
| 29 Aug | 23 Aug | 1 | 56 |
| 29 Aug | 23 Aug | 1 | 75 |
| 30 Aug | 23 Aug | 1 | 87 |
| 31 Aug | 22 Aug | 1 | 58 |
| 31 Aug | 26 Aug | 1 | 69 |
| 31 Aug | 25 Aug | 1 | 94 |
| 1 Sep | 27 Aug | 1 | 43 |
| 1 Sep | 26 Aug | 1 | 46 |
| 1 Sep | 22 Aug | 1 | 62 |
| 1 Sep | 23 Aug | 1 | 70 |
| 1 Sep | 21 Aug | 1 | 83 |
| 2 Sep | 27 Aug | 1 | 53 |
| 2 Sep | 26 Aug | 1 | 60 |
| 3 Sep | 28 Aug | 1 | 64 |
| 3 Sep | 28 Aug | 1 | 74 |
| 3 Sep | 30 Aug | 1 | 97 |
| 3 Sep | 28 Aug | 1 | 101 |
| 4 Sep | 27 Aug | 1 | 73 |
| 4 Sep | 31 Aug | 1 | 100 |
| 4 Sep | 28 Aug | 1 | 110 |
| 4 Sep | 30 Aug | 1 | 121 |
| 5 Sep | 30 Aug | 1 | 81 |
| 5 Sep | 27 Aug | 1 | 85 |
| 5 Sep | 31 Aug | 1 | 90 |
| 5 Sep | 27 Aug | 1 | 112 |
| 5 Sep | 30 Aug | 1 | 119 |
| 5 Sep | 31 Aug | 1 | 123 |
| 5 Sep | 31 Aug | 1 | 124 |
| 5 Sep | 2 Sep | 1 | 127 |
| 6 Sep | 3 Sep | 1 | 88 |
| 6 Sep | 27 Aug | 1 | 89 |
| 7 Sep | 2 Sep | 1 | 103 |
| 7 Sep | 4 Sep | 1 | 107 |
| 7 Sep | 30 Aug | 1 | 108 |
| 7 Sep | 30 Aug | 1 | 115 |
| 8 Sep | 2 Sep | 1 | 91 |
| 8 Sep | 31 Aug | 1 | 104 |
| 8 Sep | 3 Sep | 1 | 133 |
| 9 Sep | 31 Aug | 1 | 109 |
| 9 Sep | 5 Sep | 1 | 116 |
| 9 Sep | 4 Sep | 1 | 120 |
| 9 Sep | 2 Sep | 1 | 126 |
| 9 Sep | 3 Sep | 1 | 137 |
| 10 Sep | 4 Sep | 1 | 114 |
| 10 Sep | 2 Sep | 1 | 117 |
| 10 Sep | 3 Sep | 1 | 122 |
| 11 Sep | 7 Sep | 1 | 142 |
| 13 Sep | 4 Sep | 1 | 125 |
| 13 Sep | 7 Sep | 1 | 134 |
| 13 Sep | 5 Sep | 1 | 140 |
| 19 Sep | 14 Sep | 1 | 144 |
| 23 Sep | 15 Sep | 1 | 152 |
| 28 Sep | 22 Sep | 1 | 156 |
| 4 Oct | 28 Sep | 1 | 157 |
| 14 Aug | (5 Aug, 13 Aug) | 2 | 35 |
| 16 Aug | (9 Aug, 10 Aug) | 2 | 4 |
| 16 Aug | (9 Aug, 10 Aug) | 2 | 16 |
| 18 Aug | (1 Aug, 17 Aug) | 2 | 3 |
| 20 Aug | (11 Aug, 18 Aug) | 2 | 1 |
| 23 Aug | (16 Aug, 18 Aug) | 2 | 19 |
| 23 Aug | (16 Aug, 18 Aug) | 2 | 20 |
| 23 Aug | (16 Aug, 18 Aug) | 2 | 31 |
| 23 Aug | (16 Aug, 18 Aug) | 2 | 71 |
| 24 Aug | (16 Aug, 17 Aug) | 2 | 24 |
| 24 Aug | (14 Aug, 17 Aug) | 2 | 36 |
| 24 Aug | (16 Aug, 18 Aug) | 2 | 37 |
| 9 Aug | (20 Jul, 8 Aug) | 2 | 160 |
| 12 Aug | (20 Jul, 11 Aug) | 2 | 143 |
| 24 Aug | (20 Jul, 23 Aug) | 2 | 9 |
| 25 Aug | (20 Jul, 24 Aug) | 2 | 6 |
| 27 Aug | (15 Aug, 25 Aug) | 2 | 5 |
| 28 Aug | (17 Aug, 24 Aug) | 2 | 49 |
| 28 Aug | (20 Aug, 24 Aug) | 2 | 63 |
| 28 Aug | (17 Aug, 21 Aug) | 2 | 72 |
| 29 Aug | (21 Aug, 24 Aug) | 2 | 45 |
| 30 Aug | (25 Aug, 26 Aug) | 2 | 32 |
| 31 Aug | (25 Aug, 26 Aug) | 2 | 33 |
| 31 Aug | (25 Aug, 29 Aug) | 2 | 98 |
| 1 Sep | (25 Aug, 26 Aug) | 2 | 34 |
| 1 Sep | (22 Aug, 29 Aug) | 2 | 68 |
| 26 Aug | (20 Jul, 25 Aug) | 2 | 44 |
| 26 Aug | (20 Jul, 25 Aug) | 2 | 55 |
| 28 Aug | (20 Jul, 27 Aug) | 2 | 40 |
| 28 Aug | (20 Jul, 27 Aug) | 2 | 61 |
| 29 Aug | (20 Jul, 28 Aug) | 2 | 54 |
| 29 Aug | (20 Jul, 28 Aug) | 2 | 28 |
| 30 Aug | (20 Jul, 29 Aug) | 2 | 41 |
| 1 Sep | (20 Jul, 31 Aug) | 2 | 65 |
| 5 Sep | (28 Aug, 29 Aug) | 2 | 111 |
| 6 Sep | (27 Jul, 4 Sep) | 2 | 93 |
| 6 Sep | (30 Aug, 1 Sep) | 2 | 113 |
| 9 Sep | (2 Sep, 5 Sep) | 2 | 105 |
| 10 Sep | (4 Sep, 5 Sep) | 2 | 138 |
| 11 Sep | (2 Sep, 9 Sep) | 2 | 139 |
| 7 Sep | (20 Jul, 6 Sep) | 2 | 128 |
| 11 Sep | (20 Jul, 10 Sep) | 2 | 130 |
| 15 Sep | (20 Jul, 14 Sep) | 2 | 148 |
| 18 Sep | (20 Jul, 17 Sep) | 2 | 147 |
| 24 Sep | (20 Jul, 23 Sep) | 2 | 155 |
| 24 Sep | (20 Jul, 23 Sep) | 2 | 150 |
| 3 Oct | (20 Jul, 2 Oct) | 2 | 158 |
| 24 Aug | - | 3 | 2 |
| 24 Aug | - | 3 | 48 |
| 25 Aug | - | 3 | 26 |
| 29 Aug | - | 3 | 118 |
| 30 Aug | - | 3 | 27 |
| 30 Aug | - | 3 | 67 |
| 30 Aug | - | 3 | 82 |
| 30 Aug | - | 3 | 95 |
| 30 Aug | - | 3 | 102 |
| 31 Aug | - | 3 | 76 |
| 31 Aug | - | 3 | 80 |
| 31 Aug | - | 3 | 86 |
| 1 Sep | - | 3 | 42 |
| 1 Sep | - | 3 | 66 |
| 2 Sep | - | 3 | 96 |
| 4 Sep | - | 3 | 92 |
| 4 Sep | - | 3 | 99 |
| 5 Sep | - | 3 | 84 |
| 5 Sep | - | 3 | 129 |
| 10 Sep | - | 3 | 132 |
| 10 Sep | - | 3 | 135 |
| 12 Sep | - | 3 | 131 |
| 13 Sep | - | 3 | 145 |
| 15 Sep | - | 3 | 146 |
| 18 Sep | - | 3 | 141 |
| 22 Sep | - | 3 | 151 |
| 23 Sep | - | 3 | 149 |
| 24 Sep | - | 3 | 153 |
| 29 Sep | - | 3 | 154 |
| 7 Oct | - | 3 | 159 |
