# Supplementary material for: Assessing dengue control in Tokyo, 2014
Source: PLoS Negl Trop Dis. 2019 Jun 21;13(6):e0007468. doi: 10.1371/journal.pntd.0007468 (PMC6588210; doi:10.1371/journal.pntd.0007468)
Supplement: S2 Table — (DOCX) [file pntd.0007468.s004.docx]

## S2 Table. Comparison of model fit by the number of generations and time lag between infection in the unobserved index case and observed first exposure (4 August 2014)

| *n* | *d*_0_ | *μ*_IP_ | *σ*_IP_ | *μ*_Trans_ | *σ*_Trans_ | *R*_1_ | *R*_2_ | *R*_3_ | *ε*_1_ | *ε*_2_ | AIC |
| --- | --- | --- | --- | --- | --- | --- | --- | --- | --- | --- | --- |
| 2 | 3 | 5.8 | 1.9 | 8.7 | 7.4 | 7.0 | - | - | 0.9 | 0.7 | 1858.7 |
| 2 | 4 | 5.8 | 1.9 | 9.4 | 7.4 | 7.5 | - | - | 0.8 | 0.7 | 1856.1 |
| 2 | 5 | 5.8 | 1.8 | 10.0 | 7.3 | 8.1 | - | - | 0.8 | 0.7 | 1854.9 |
| 2 | 6 | 5.8 | 1.8 | 10.8 | 7.3 | 8.7 | - | - | 0.7 | 0.6 | 1854.4 |
| 2 | 7 | 5.8 | 1.8 | 11.3 | 7.2 | 9.3 | - | - | 0.7 | 0.6 | 1854.3 |
| 2 | 8 | 5.8 | 1.8 | 11.9 | 7.2 | 10.0 | - | - | 0.6 | 0.6 | 1854.5 |
| 2 | 9 | 5.8 | 1.8 | 12.5 | 7.2 | 10.7 | - | - | 0.6 | 0.6 | 1854.8 |
| 3 | 3 | 5.8 | 1.9 | 5.5 | 5.4 | 3.8 | 2.8 | - | 0.5 | 0.5 | 1860.0 |
| 3 | 4 | 5.8 | 1.9 | 8.6 | 6.6 | 7.2 | 0.1 | - | 0.9 | 0.7 | 1858.1 |
| 3 | 5 | 5.8 | 1.8 | 8.7 | 5.9 | 7.3 | 0.2 | - | 0.8 | 0.7 | 1856.6 |
| 3 | 6 | 5.8 | 1.8 | 9.0 | 5.5 | 7.7 | 0.2 | - | 0.8 | 0.7 | 1855.7 |
| 3 | 7 | 5.8 | 1.8 | 9.3 | 5.2 | 8.0 | 0.2 | - | 0.8 | 0.8 | 1855.3 |
| 3 | 8 | 5.8 | 1.8 | 9.8 | 5.0 | 8.3 | 0.2 | - | 0.8 | 0.8 | 1855.0 |
| 3 | 9 | 5.8 | 1.8 | 10.2 | 4.8 | 8.7 | 0.2 | - | 0.8 | 0.8 | 1855.0 |
| 4 | 3 | 5.8 | 1.9 | 5.0 | 4.2 | 4.3 | 2.1 | 0.2 | 0.5 | 0.5 | 1861.8 |
| 4 | 4 | 5.8 | 1.9 | 5.3 | 3.3 | 5.0 | 1.8 | 0.3 | 0.5 | 0.5 | 1859.2 |
| 4 | 5 | 5.8 | 1.8 | 5.6 | 2.7 | 5.3 | 2.0 | 0.4 | 0.5 | 0.4 | 1857.0 |
| 4 | 6 | 5.8 | 1.8 | 5.9 | 2.3 | 5.3 | 2.5 | 0.4 | 0.4 | 0.4 | 1855.2 |
| 4 | 7 | 5.8 | 1.9 | 6.2 | 2.1 | 5.3 | 3.4 | 0.4 | 0.3 | 0.4 | 1854.1 |
| 4 | 8 | 5.8 | 1.9 | 6.6 | 2.0 | 5.3 | 4.4 | 0.4 | 0.3 | 0.4 | 1854.0 |
| 4 | 9 | 5.8 | 1.8 | 7.6 | 2.3 | 6.6 | 1.1 | 0.3 | 0.9 | 0.3 | 1855.7 |

*n* represents the number of generations for generation-dependent model (i.e., there are in total *n*+1 generations including generation zero). *d*_0_ defines lag days required from the actual start of exposure to the first reported date of exposure on 4 August 2014. Mean *μ*_IP_ and standard deviation *σ*_IP_ of the incubation period were assumed as the parameters governing the gamma distribution. Mean *μ*_Trans_ and standard deviation *σ*_Trans_ of the waiting time for the generation time other than incubation period were also assumed to follow a gamma distribution. *R*_n-1_ stands for the reproduction number of the *n*^th^-generation infection in the absence of interventions. To account for the effectiveness of interventions, *ε*_i_ is factored in our mathematical model to represent the relative reduction in the reproduction number due to mosquito control (*ε*_1_) and park closure (*ε*_2_). AIC stands for the Akaike information criterion.
