# Supplementary material for: Assessing dengue control in Tokyo, 2014
Source: PLoS Negl Trop Dis. 2019 Jun 21;13(6):e0007468. doi: 10.1371/journal.pntd.0007468 (PMC6588210; doi:10.1371/journal.pntd.0007468)
Supplement: S3 Table — (DOCX) [file pntd.0007468.s005.docx]

## S3 Table. Comparison of model fit by the number of generations and four settings of hypothesized effectiveness measures.

|  | Parameters | Setting 1  (*ε*_1_ ≠1 and *ε*_2_≠1) | Setting 2  (*ε*_1_=1 and *ε*_2_≠1) | Setting 3  (*ε*_1_ ≠1 and *ε*_2_=1) | Setting 4  (*ε*_1_=1 and *ε*_2_=1) |
| --- | --- | --- | --- | --- | --- |
| Two-generation model (G2) | *d*_0_ | 7 | 7 | 7 | 7 |
|  | *μ*_IP_ | 5.8 | 5.8 | 5.8 | 5.8 |
|  | *σ*_IP_ | 1.8 | 1.8 | 1.8 | 1.8 |
|  | *μ*_Trans_ | 11.4 | 11.4 | 11.4 | 11.4 |
|  | *σ*_Trans_ | 7.3 | 7.3 | 7.3 | 7.3 |
|  | *R*_0_ | 15.1 | 15.1 | 15.1 | 15.1 |
|  | *R*_1_ | 9.4 | 7.2 | 9.3 | 4.8 |
|  | *R*_2_ | - | - | - | - |
|  | *R*_3_ | - | - | - | - |
|  | *ε*_1_ | 0.7 | 1.0 | 0.5 | 1.0 |
|  | *ε*_2_ | 0.6 | 0.5 | 1.0 | 1.0 |
|  | AICc | 1855.1 | 1858.7 | 1858.5 | 1871.6 |
| Three-generation model (G3) | *d*_0_ | 9 | 9 | 9 | 9 |
|  | *μ*_IP_ | 5.8 | 5.8 | 5.8 | 5.8 |
|  | *σ*_IP_ | 1.8 | 1.8 | 1.8 | 1.8 |
|  | *μ*_Trans_ | 10.3 | 10.3 | 10.3 | 10.3 |
|  | *σ*_Trans_ | 4.9 | 4.9 | 4.9 | 4.9 |
|  | *R*_0_ | 13.6 | 13.6 | 13.6 | 13.6 |
|  | *R*_1_ | 8.8 | 7.6 | 8.8 | 7.3 |
|  | *R*_2_ | 0.2 | 0.2 | 0.1 | 0.1 |
|  | *R*_3_ | - | - | - | - |
|  | *ε*_1_ | 0.7 | 1.0 | 0.7 | 1.0 |
|  | *ε*_2_ | 0.8 | 0.7 | 1.0 | 1.0 |
|  | AICc | 1856.0 | 1858.0 | 1856.4 | 1859.5 |
| Four-generation model (G4) | *d*_0_ | 8 | 8 | 8 | 8 |
|  | *μ*_IP_ | 5.8 | 5.8 | 5.8 | 5.8 |
|  | *σ*_IP_ | 1.9 | 1.9 | 1.9 | 1.9 |
|  | *μ*_Trans_ | 6.6 | 6.6 | 6.6 | 6.6 |
|  | *σ*_Trans_ | 2.0 | 2.0 | 2.0 | 2.0 |
|  | *R*_0_ | 4.0 | 4.0 | 4.0 | 4.0 |
|  | *R*_1_ | 5.4 | 6.1 | 5.5 | 6.5 |
|  | *R*_2_ | 4.3 | 1.0 | 4.0 | 0.6 |
|  | *R*_3_ | 0.4 | 0.4 | 0.2 | 0.2 |
|  | *ε*_1_ | 0.3 | 1.0 | 0.2 | 1.0 |
|  | *ε*_2_ | 0.4 | 0.4 | 1.0 | 1.0 |
|  | AICc | 1855.2 | 1858.0 | 1865.1 | 1864.9 |

Note: Setting 1 (*ε*_1_≠1 and *ε*_2_≠1): two effectiveness measures are jointly estimated. Setting 2 (*ε*_1_=1 and *ε*_2_≠1): only the park closure effect is estimated. Setting 3 (*ε*_1_ ≠1 and *ε*_2_=1): only mosquito control and public awareness campaigns factored in. Setting 4 (*ε*_1_=*ε*_2_=1): no effects of control measures are taken into account.
