# Supplementary material for: Assessing dengue control in Tokyo, 2014
Source: PLoS Negl Trop Dis. 2019 Jun 21;13(6):e0007468. doi: 10.1371/journal.pntd.0007468 (PMC6588210; doi:10.1371/journal.pntd.0007468)
Supplement: S1 Text — (DOCX) [file pntd.0007468.s006.docx]

## S1 Text. Derivation of the generation-dependent model.

To introduce the generation-dependent model, we first use the renewal equation

$i(t)=R(t)\int_{0}^{\infty} i(t-\tau)g(\tau)d\tau$, (S1)

where $i(t)$ represents the incidence at time *t*, $R(t)$ is the effective reproduction number, and $g(\tau)$ is the probability density function of generation time. Let $i_{m}(t)$ be the incidence of generation *m* at time *t*, $i(t)$ can be the total number of cases over different generations *m* at time *t* as

$i(t)=\sum_{m=1}^{n} i_{m}(t)$, (S2)

where *n* represents the maximum number of generations.

Moreover, $i_{m}(t)$ can be rewritten from equation (A1) as

$i_{m}(t)=R_{m-1}\int_{0}^{\infty} i_{m-1}(t-\tau)g(\tau)d\tau$, (S3)

where $R_{m-1}$ is the reproduction number of generation *m*. If we substitute equation (S3) into (S2) and discretize it, $i(t)$ can be derived as

$i\left( t \right)= R_{0}\left( g_{t}+R_{1}\left( g*g \right)_{t}+R_{2}R_{1}\left( g*g*g \right)_{t}+R_{3}R_{2}R_{1}\left( g*g*g*g \right)_{t} \right)$. (S4)

Equation (S4) is the numerator of equation (2) in the main text, with $n=4$.
